# Antenatal Ultrasound Utilization and Associated Factors Among Postpartum Women in Durame Town Public Health Institutions, Central Ethiopia: A Facility‐Based Cross‐Sectional Study

**DOI:** 10.1155/tswj/5637805

**Published:** 2026-08-04

**Authors:** Abdulaziz Assefa, Aynalem Belay, Mulugeta Animaw, Mangistu Abera, Fikremariam Endeshaw, Seid Jemal, Ayele Sahile Abdo, Aberash Beyene Derribow, Yohannes Fikadu Geda

**Affiliations:** ^1^ Department of Midwifery, College of Medicine and Health Sciences, Wolkite University, Wolkite, Ethiopia, wku.edu.et; ^2^ Faculty of Health, Charles Darwin University, Darwin, Australia, cdu.edu.au

**Keywords:** antenatal ultrasound, Durame, Ethiopia, postnatal women

## Abstract

**Background:**

Antenatal ultrasound scanning is a fundamental component of antenatal care across the globe. It is of significant public health importance because it enables early detection of fetal abnormalities, monitors fetal growth and maternal health, informs timely medical interventions, and supports strategies to reduce maternal and neonatal morbidity and mortality. Despite its clinical importance, there are limited studies on the use of antenatal ultrasound in developing countries, including Ethiopia.

**Objective:**

This study is aimed at assessing the antenatal ultrasound utilization and associated factors among postpartum mothers in Durame Town public health institutions, Central Ethiopia, 2025.

**Methods:**

A facility‐based cross‐sectional study was conducted on 379 postpartum mothers attending postpartum care from September 10 to November 10, 2025, in Durame Town public health institutions. A systematic random sampling technique was used to select study participants. Data were collected using structured and pretested questionnaires and analyzed by SPSS Version 26. Factors associated with antenatal ultrasound utilization among postpartum mothers were assessed using binary logistic regressions. The strength of the association of dependent and independent variables was presented as crude and adjusted odds ratios (AORs) at a 95% confidence interval. The level of significance was declared at a *p* value of less than 0.05 in multivariable logistic regression.

**Results:**

Based on the present study, the proportion of antenatal ultrasound utilization was 249 (65.7%) (95% CI: 60.9–70.5); 46.2% of mothers had early antenatal ultrasound utilization, done before they were 24 weeks. Factors associated with antenatal ultrasound utilization were living in urban residence (AOR = 3.25; 95% CI: 1.55–5.65), previous delivery with a congenital malformation (AOR = 2.906; 95% CI: 1.222–6.91), and mothers′ knowledge on antenatal ultrasound utilization (AOR = 3.132; 95% CI: 1.425–6.88).

**Conclusion and Recommendation:**

Based on the present study, the proportion of antenatal ultrasound utilization was lower than the WHO and FIGO recommendations. Significant factors of antenatal ultrasound utilization were residence, women′s knowledge, and congenital malformation. Therefore, healthcare providers better to enhance awareness and education among pregnant women regarding the benefits of antenatal ultrasound services, whereas policymakers and health facility administrators better to improve service accessibility and availability to increase antenatal ultrasound utilization, especially in rural and underserved areas, and pay special attention to mothers with prior adverse pregnancy outcomes or congenital malformations.

## 1. Introduction

Obstetric ultrasound is a harmless, cheap, and noninvasive imaging modality that helps to scan a pregnant mother′s abdominal and pelvic cavity delivers parents with a real‐time image of the fetus [1]. Antenatal ultrasound utilization is an integral part of antenatal care (ANC) around the world [2].

Antenatal ultrasound has become a cornerstone of modern ANC, providing significant information for pregnancy monitoring and enhancing maternal and neonatal outcomes. It is widely used to confirm pregnancy, estimate gestational age, identify multiple pregnancies, assess fetal growth, and detect congenital anomalies early, thereby guiding timely interventions and management strategies [1, 2].

Despite the verified benefits, globally, disparities in antenatal ultrasound utilization are evident between high‐ and low‐income countries. In high‐income countries, utilization of ultrasound exceeds 90%, reflecting availability, awareness, and integration into routine ANC [3]. In contrast, resource‐limited settings report far lower rates, often below 50%, due to limited infrastructure, shortages of trained personnel, financial barriers, and sociocultural influences [4]. A study in sub‐Saharan Africa revealed that less than half of pregnant women received at least one antenatal ultrasound during pregnancy, contributing to missed opportunities for early detection of complications [3].

In Ethiopia, although ANC coverage has enhanced in recent years, antenatal ultrasound utilization remains insufficient and inequitably distributed. The 2024–25 Ethiopian Demographic and Health Survey (EDHS) reported that only 78% of pregnant women received ANC from skilled providers [5]. There were no national data on the proportion of antenatal ultrasound utilization in Ethiopia. However, some recent facility‐based studies have revealed varying utilization. For instance, studies conducted in South Wollo, Addis Ababa, and Jimma, where the proportion of antenatal ultrasound utilization was 60.7% [6], 70.3% [7], and 62.8% [8], respectively.

Antenatal ultrasound utilization is influenced by various factors, including maternal sociodemographic characteristics, awareness, cultural beliefs, accessibility of health services, and provider‐related barriers [6, 9]. Studies have shown variation between urban and rural areas, with rural women often facing limited access due to distance, cost, and shortage of trained personnel [10]. Recent studies undertaken in different regions of Ethiopia show significant variation, with usage ranging from 25% to 65%, revealing differences in health service access, socioeconomic status, and maternal education [7, 8, 11]. Moreover, structural obstacles, including inadequate ultrasound machines, shortage of trained sonographers, and weak referral systems, limit effective use of this service [12].

The World Health Organization (WHO) recommends at least one ultrasound scan earlier than 24 weeks of gestation to strengthen pregnancy outcomes by supporting early detection of complications [13]. Likewise, the International Federation of Gynecology and Obstetrics (FIGO) recommends two antenatal ultrasound scans for all pregnant women during the first and second trimesters [14]. Moreover, the Society of Obstetricians and Gynaecologists of Canada suggests offering all pregnant women an ultrasound scan between Weeks 18 and 22 to identify potential fetal congenital malformations [15].

The Ethiopia Ministry of Health endorses that all pregnant women employ antenatal ultrasound scans timely (before 24 weeks of gestation) as routine ANC [16]. Antenatal ultrasound utilization is not only fundamental for personal care but also has wider public health importance, especially in achieving the Sustainable Development Goals (SDGs). For instance, antenatal ultrasound services help early identification of high‐risk pregnancies and complications, prompt management, or referral, contributing to improved maternal and neonatal health, which is an aim central to SDG 3 on ensuring healthy lives and well‐being for all. In Ethiopia, evidence indicates that enhancing antenatal ultrasound services at primary health centers played a role in averting a substantial number of maternal and neonatal morbidities and mortalities [17].

In Ethiopia, despite sustainable efforts to improve maternal health services, studies reveal that countless expectant pregnant women still have inadequate utilization of antenatal ultrasound during ANC visits due to both challenges from the demand and supply perspectives [8]. Therefore, despite the documented medical benefits of antenatal ultrasound, gaps in usage remain across global, national, and local contexts. Assessing the extent of antenatal ultrasound utilization and associated factors in this context is crucial to inform scientifically validated interventions, strengthen maternal health outcomes, and conform to national strategies to reduce maternal and neonatal mortality.

To the best of the authors′ knowledge, there is no evidence about the utilization of antenatal ultrasound in Ethiopia, particularly in the study area (Public Health Institutions of Durame Town). Therefore, this study is aimed at assessing the antenatal ultrasound utilization and associated factors among postpartum women in Public Health Institutions of Durame Town, Central Ethiopia, 2025.

## 2. Methods

### 2.1. Study Design, Area, and Period

A facility‐based cross‐sectional study was conducted at Durame Town public health institutions from September 10 to November 10, 2025. The town is the administrative center of the Kembata Tembaro Zone, Central Ethiopia region. It is 352 km away from Addis Ababa, the capital city of Ethiopia, and 125 km from Hawassa, capital city of the region, respectively. The town has three public health centers and one public hospital (Durame General Hospital). The hospital provides services for the Durame Town population and for all neighboring district populations. Durame General Hospital (MCH) Center provides various services with its department of surgery, gynecology, pediatrics, medical, obstetrics, emergency, laboratory, pharmacy, and ART clinic. At the health centers, postpartum women from the nearby urban and rural communities are seen, and primary health care services (including ANC, delivery services, postpartum care, and family planning) to all population groups are provided. Based on the past 3 months′ statistics, the estimated average number of pregnant women who attended ANC and gave birth in selected Durame Town public health institutions was 435 and 402 per month, respectively.

### 2.2. Study Participants

All postpartum women attending postnatal care services in public health institutions of Durame Town were the source population of the study, whereas postpartum women who were available during the data collection period were considered as the study population.

### 2.3. Inclusion and Exclusion Criteria

#### 2.3.1. Inclusion Criteria

Postpartum mothers who attended PNC in public health institutions of Durame Town within the first 7 days following childbirth are included.

#### 2.3.2. Exclusion Criteria

Postpartum mothers who cannot recall antenatal ultrasound utilization or incomplete files on ultrasound utilization during their recent pregnancy and are seriously ill and unable to respond to questions during the data collection period are excluded.

### 2.4. Sample Size and Sampling Procedures

#### 2.4.1. Sample Size

The sample size was calculated using a single population proportion formula with the following assumptions:

n=zα/22p1−pd2,

where *n* is the minimum sample size needed, *p* is the estimated proportion of prenatal ultrasound utilization, *z* is the standard value of the confidence level of *α* = 95*%*, and d = 0.05 is the margin of error between the sample and the population. For this study, *p* = 60.7*%*, since a study was conducted in Jimma, Oromia Region, Ethiopia, on the proportion of prenatal ultrasound utilization [8].

p=0.60721.960.05366.4,za/=,d=,n=0.6070.3930.052,n=.

By using a 10% nonresponse rate, the final sample size is 366.4 + 36.4 = 403.

#### 2.4.2. Sampling Procedures and Technique

A systematic random sampling technique was employed to select the study participants. Three public health centers and one public hospital (Durame General Hospital) were found in the town. One public health center and one general hospital were purposively selected based on the availability of data. The allocation of the sample to health facilities was performed proportionally based on the average number of clients who received postnatal care at each facility in the month preceding the data collection period. The respondents were selected in the order in which they came to health facilities. The average 2‐month reported number of postpartum clients was approximately 805.

Participants′ card numbers were used to systematically select study participants every K interval (taking K = N/n = 805/403 ≈ 2 [where N is the source population, and n is the sample size for this study]). A random number between 1 and 2 was selected using the lottery method to determine the starting point. Therefore, the actual data collection was started from the first mother. Subsequently, every second woman arriving at the postpartum clinic was invited to participate. For any selected woman who was ineligible or refused to participate, the next consecutive woman was considered to maintain the interval.

### 2.5. Study Variables

#### 2.5.1. Dependent Variable

The dependent variable is antenatal ultrasound utilization.

#### 2.5.2. Independent Variables

The independent variables are as follows:

Sociodemographic factors: age, residence, marital status, religion, ethnicity, educational status, occupation, and average monthly income.

Obstetric‐related factors: gravidity, parity, gestational age, trimester of pregnancy, number of ANC contacts, and place of delivery, like bad obstetric history.

Knowledge‐related factors: knowledge of postpartum mothers on the importance of antenatal ultrasound utilization.

### 2.6. Operational Definitions

Antenatal ultrasound utilization refers to the use of obstetric ultrasonography at least once during pregnancy for diagnostic or monitoring purposes, such as pregnancy dating, detecting multiple gestations, assessing fetal growth, screening for anomalies, and monitoring complications. In this study, utilization is considered “yes” if the mother received at least one ultrasound scan during her current pregnancy and “no” if she did not receive any scan during ANC contacts [18].

Knowledge of antenatal ultrasound: Respondents who answered greater than 50% of the knowledge questions were considered as having good knowledge, whereas the rest were considered as having poor knowledge [19].

Incomplete files on ultrasound utilization: refers to situations where a participant′s information regarding whether she underwent antenatal ultrasound and/or the timing of the examination (early or late) was incomplete or could not be verified from her medical record, particularly in cases where the woman was unable to recall whether she had undergone antenatal ultrasound and there was no documented evidence in her file to confirm it [20].

### 2.7. Data Collection Method and Instrument

The data collection tool was adapted by reviewing related literature and modified according to the local situation of the study area [6–8, 21–23]. Data were collected using a structured and pretested questionnaire through face‐to‐face interviews and chart review after the study participants received postpartum care services. The tools included sociodemographic variables, obstetric‐related factors, and mothers′ knowledge of antenatal ultrasound.

### 2.8. Data Quality Control Management

The quality of data was assured before data collection, during data collection, and after data collection. The tool was first prepared in English, then translated into Amharic, and translated again into English by a language translator expert who is fluent in both languages and familiar with the study context to ensure internal consistency. Amharic might not be the mother tongue for all participants in the study setting. However, it is widely used as a working language in healthcare settings and is commonly understood by most clients attending the selected health facilities. To ensure inclusiveness and accurate understanding, data collectors were trained to administer the questionnaire in the local language, when necessary, and to clarify any items for participants who had difficulty understanding Amharic.

A pretest was conducted on 5% [21] of the sample size from Halaba General Hospital, and the group leader provided a 1‐day training and discussion for the data collectors before the data collection process. During data collection, the group leader performed close follow‐up. After data collection, data were checked for completeness, and data cleaning was conducted before analysis to identify and resolve outliers and inconsistencies using SPSS.

### 2.9. Data Processing and Analysis

Data were cleaned, coded, and entered using EpiData Version 4.6.0.0 software, and the data were crosschecked for completeness before analysis. The entered data were exported and analyzed with SPSS Version 26. Logistic regression was used to analyze the associations between the independent variables and the outcome of antenatal ultrasound utilization. The final model′s goodness‐of‐fit was checked by Hosmer and Lemeshow′s test, and the model was fit with a *p* value of 0.469. Variables were assessed for multicollinearity, and the data met the assumption of collinearity with a variance inflation factor of 1.13–4.12 and a tolerance test of 0.20–0.89. Variables with a *p* value < 0.25 in the bivariate analyses were used as the cut point for eligibility in the multivariable binary logistic regression model. Finally, statistically significant variables were declared at *p* value < 0.05and were reserved in the final model with 95% CI.

## 3. Result

### 3.1. Sociodemographic Characteristics

In this study, 379 mothers were interviewed with a response rate of 94.04%. The mean age ± SD of the participants was 28.2 ± 6.60 years, and more than half of respondents, 213 (56.2%), were between 20 and 29 years old.

More than half of the participants, 217 (57.3%), were urban residents, and nearly one‐third of participants, 111 (29.3%), had a college diploma or higher. More than two‐thirds of the women (67.2%) were housewives and partly working in public or private businesses, and more than half (58%) were Protestant, followed by Orthodox (27.2%); 260 (68.6%) were Kembata in ethnic background (Table 1).

**Table 1:** Sociodemographic characteristics of postpartum women at Durame Town public health institutions, Central Ethiopia, 2025.

Variable	Category	Frequency (*N*)	Percentage (%)
Age (years)	< 20	22	5.8
	20–29	213	56.2
	30–39	128	33.8
	≥ 40	16	4.2
Marital status	Married	367	96.8
	Single	4	1.1
	Divorced	8	2.1
Educational status (self)	College diploma and above	111	29.3
	Secondary	102	26.9
	Primary	67	17.7
	Read and write	60	15.8
	Unable to read and write	39	10.3
Educational status (husband)	College diploma and above	162	42.7
	Secondary	48	12.7
	Primary	97	25.6
	Read and write	28	7.4
	Unable to read and write	44	11.6
Residence	Urban	217	57.3
	Rural	162	42.7
Religion	Orthodox	103	27.2
	Muslim	24	6.3
	Catholics	32	8.4
	Protestant	220	58.0
Ethnicity	Kembata	260	68.6
	Hadiya	45	11.9
	Sidama	32	8.4
	Silte	24	6.3
	Wolayita	18	4.7
Occupation	Housewife	255	67.3
	Government employee	16	4.2
	Private employee	69	18.2
	Student	39	10.3
Monthly income (ETB)	< 5000	106	28.0
	5000–10,000	157	41.4
	10,001–15,000	79	20.8
	> 15,000	37	9.8

### 3.2. Obstetric‐Related Characteristics

In the present study, three‐quarters of participants, 286 (75.5%), had been pregnant between two and four times. Nearly one‐third, 117 (30.9%), of the mothers had experienced abortion, and 281 (74.1%) of the mothers had no history of medical illness during their most recent pregnancy (Table 2).

**Table 2:** Obstetrical characteristics of postpartum women at Durame Town public health institutions, Central Ethiopia, 2025.

Variable	Category	Frequency (*N*)	Percentage (%)
Gravidity (no. of pregnancies)	Primigravida	93	24.5
	Multigravida	286	75.5
Parity (no. of births)	Primiparous	105	27.7
	Multiparous	274	72.3
History of abortion	Yes	117	30.9
	No	262	69.1
Frequency of ANC visits	< 4	167	44.1
	≥ 4	212	55.9
History of ectopic pregnancy	Yes	28	7.4
	No	351	92.6
Medical illness during recent pregnancy	Yes	98	25.9
	No	281	74.1
Congenital malformation	Yes	26	6.9
	No	353	93.1

### 3.3. Knowledge of Mothers on Ultrasound Utilization

Based on the present study, 221 (67.5%) (95% CI: 66.0–69.0) of postpartum mothers had good knowledge of antenatal ultrasound utilization. The most common knowledge component reported by study participants was helping to confirm the presence of multiple pregnancies, which was reported by approximately 358 (94.5%) mothers. Estimating gestational age was the second most reported importance of ultrasound, with 335 (88.4%) reporting it. The least common component of knowledge regarding antenatal ultrasound was detecting pregnancy complications, which was reported by only 68 (17.9%) participants (Table 3).

**Table 3:** Knowledge of mothers on antenatal ultrasound utilization among postpartum mothers at Durame Town public health institutions, Central Ethiopia, 2025.

Variables	Categories	Frequency	Percentage
Does antenatal ultrasound assess the well‐being of the fetus?	Yes	244	64.4
	No	135	35.6
Does antenatal ultrasound confirm presence of multiple pregnancies?	Yes	358	94.5
	No	21	5.5
Does antenatal ultrasound estimate gestational age?	Yes	335	88.4
	No	44	11.6
Does antenatal ultrasound estimate fetal weight?	Yes	210	65.4
	No	169	44.6
Does antenatal ultrasound confirm pregnancy?	Yes	322	85.0
	No	57	15.0
Does antenatal ultrasound detect pregnancy complications?	Yes	68	17.9
	No	311	82.1
Does antenatal ultrasound detect amniotic fluid volume?	Yes	282	74.4
	No	97	25.6
Does antenatal ultrasound determine the expected date of delivery (EDD)?	Yes	268	70.7
	No	111	29.3
Does antenatal ultrasound detect defects or congenital abnormalities?	Yes	264	69.7
	NO	115	30.3
Does antenatal ultrasound determine cord and placenta position?	Yes	206	54.4
	No	173	45.6

### 3.4. Antenatal Ultrasound Utilization

Based on the present study, the proportion of antenatal ultrasound utilization was 249 (65.7%) (95% CI: 60.9–70.5). Less than half, 115 (46.2%), of participants had early antenatal ultrasound utilization, which is the first ultrasound done before they were 24 weeks. A total of 115 (42.2%) of the women had an antenatal ultrasound scan requested by midwives or nurses, and (40.6%) had only one antenatal ultrasound scan in their recent pregnancy (Table 4).

**Table 4:** Antenatal ultrasound utilization among postpartum mothers at Durame Town public health institutions, Central Ethiopia, 2025.

Variable	Category	Frequency (*N*)	Percentage (%)
Used ultrasound in recent pregnancy	Yes	249	65.7
	No	130	34.3
Timing of first ultrasound	Early (before 6 months/< 24 weeks)	115	46.2
	Late (after 6 months/> 24 weeks)	134	53.8
Who suggested the ultrasound?	Midwife or nurse	105	42.2
	Doctor	84	33.7
	Self or family decision	36	14.5
	Not remember	24	9.6
Number of scans in recent pregnancy	Only one	101	40.6
	2–3 times	89	35.7
	4–5 times	36	14.5
	More than 5 times	23	9.2

### 3.5. Factors Associated With Utilization of Ultrasound

In order to identify the associated factors of antenatal ultrasound utilization, variables with a *p* value of less than 0.25 in bivariable binary logistic regression analysis were included in the multivariable model. Seven variables (residence, maternal educational status, parity, previous history of abortion, previous congenital malformation, medical illness during pregnancy, and knowledge on ultrasound utilization) were entered into the multivariable logistic regression model. Only three variables (residence, knowledge of mothers on ultrasound, and history of congenital malformation) were significant variables with antenatal ultrasound utilization.

In the present study, mothers who were living in urban areas were 3.25 times more likely to utilize antenatal ultrasound than mothers who reside in rural areas (AOR = 3.25; 95% CI: 1.55–5.65).

Furthermore, mothers who had good knowledge of ultrasound utilization were 3.13 times more likely to utilize antenatal ultrasound compared with those with poor knowledge of ultrasound utilization (AOR = 3.132; 95% CI: 1.425–6.88).

Another significant associated factor was mothers who had a previous delivery with a congenital malformation were nearly three times more likely to have obstetric antenatal ultrasound examination (AOR = 2.906; 95% CI: 1.222–6.91) (Table 5).

**Table 5:** Bivariable and multivariable logistic regression analysis to assess the antenatal ultrasound utilization and associated factors among postpartum mothers in Durame Town public health institutions, Central Ethiopia, 2025.

Ultrasound utilization					
Variables	Yes *n* (%)	No *n* (%)	COR (95% CI)	AOR (95% CI)	*p*
Residence					
Urban	175 (80.6)	42 (19.4)	1	1	
Rural	71 (25.3)	91 (74.7)	5.34 (1.01–7.17)	**3.25 (1.55–5.65)**∗	**0.003**
Maternal educational level					
Unable to read and write	25 (64.1)	14 (35.9)	0.82 (0.35, 4.97)	0.48 (0.19, 4.210)	0.67
Read and write	40 (66.7)	20 (33.3)	0.73 (0.251, 2.46)	0.56 (0.29, 6.01)	0.89
Primary education	46 (68.7)	21 (31.3)	0.67 (0.09, 3.33)	0.53 (0.15, 9.36)	0.09
Secondary education	61 (59.8)	41 (40.2)	0.99 (0.18, 2.92)	0.29 (0.01, 3.92)	0.95
Collage and above	66 (59.5)	45 (40.5)	1	1	
Parity					
Primiparous	80 (80.8)	19 (19.2)	1	1	
Multiparous	209 (76.6)	64 (23.4)	1.29 (0.44, 3.37)	1.43 (0.78, 2.62)	0.384
History of abortion					
No	160 (61)	102 (39)	1	1	
Yes	79 (67.5)	39 (32.5)	0.78 (0.13, 2.68)	0.32 (0.12, 5.91)	0.10
Previous congenital malformation					
No	282 (79.9)	71 (20.1)	1	1	
Yes	14 (53.8)	12 (46.2)	3.39 (1.13, 5.68)	**2.906 (1.22, 6.91)**∗	**0.002**
Medical illness during pregnancy					
No	224 (79.7)	57 (20.3)	1	1	
Yes	72 (73.5)	26 (26.5)	1.42 (0.41, 4.21)	1.02 (0.43, 5.35)	0.198
Women′s knowledge on ultrasound					
Poor	18 (52.9)	16 (47.1)	1	1	
Good	278 (80.6)	67 (19.4)	0.27 (0.71, 7.53)	**3.132 (1.43, 6.88)**∗	**0.001**

*Note:* Key 1 = reference. Asterisk “∗” denotes statistically significant at 95% CI, *p* < 0.05 antenatal ultrasound utilization.

Bold values indicate statistically significant variables.

Abbreviations: AOR, adjusted odds ratio; CI, confidence interval; COR, crude odds ratio.

## 4. Discussion

Antenatal ultrasound utilization has wider public health importance, especially in achieving the SDGs. According to the recommendations from FIGO, WHO, and FMOH, all pregnancies should undergo a minimum of two scans throughout pregnancy. This study is aimed at assessing antenatal ultrasound utilization and associated factors among postpartum mothers at Durame Town public health institutions, Central Ethiopia, in 2025.

Based on the present study, the proportion of antenatal ultrasound utilization was 65.7% (95% CI: 60.9–70.5), 46.2% of mothers had early antenatal ultrasound utilization, done before they were 24 weeks. The finding of this study is congruent with studies conducted at South Wollo [6], Addis Ababa [7], and Jimma [8], indicating relatively uniform antenatal ultrasound utilization across these contexts.

However, the finding of the current study was lower than the retrospective study conducted in Ndejje Health Centre (Uganda), where for 105 singleton deliveries, mothers underwent 232 obstetric scans [24]. Moreover, the finding of the present study is also lower than the studies conducted at Zaria, Kaduna State, Northern Nigeria [25], Eastern China [26], and the United Kingdom [27]. The possible discrepancy might be due to sociodemographic differences, variations in the sample size, and the health service system between Ethiopia and the above countries. For example, women with higher education or living in urban settings are more likely to attend regular antenatal visits and undergo the recommended number of ultrasound scans, whereas women with lower education, lower income, or residing in rural settings may face obstacles that hinder their access to antenatal ultrasound scan.

In contrast, the finding of the present study was higher than studies conducted in Southeastern Nigeria [28] and Kenya [21]. This discrepancy may be attributed to variations in health service availability, maternal health policies, and levels of awareness and access to ANC services across the study settings.

The finding of the current study showed that there was a strong association between participants′ residence and the utilization of antenatal ultrasound. Mothers living in urban areas were 3.25 times more likely to utilize antenatal ultrasound than mothers living in rural areas (AOR = 3.25; 95% CI: 1.55–5.65). The finding of this study is in line with studies conducted at South Wollo [6] and Jimma [8], where participants′ residence was significantly associated with antenatal ultrasound utilization. This may be attributed to better access to information and greater awareness of antenatal ultrasound services among women in urban areas compared with those in rural settings, as well as differences in proximity to health facilities. Additionally, limited infrastructure development in rural parts of the country may further reduce access to and utilization of antenatal ultrasound services, as supported by previous studies [29].

Furthermore, mothers who had good knowledge of ultrasound utilization were 3.13 times more likely to utilize antenatal ultrasound compared with those with poor knowledge of ultrasound utilization (AOR = 3.132; 95% CI: 1.425–6.88). The finding of this study is congruent with studies conducted in Sokoto, Nigeria [28], Uganda [24], South Wollo [6], and Jimma [8], where a mother′s knowledge of ultrasound was significantly associated with antenatal ultrasound utilization. This may be because mothers with good knowledge of antenatal ultrasound understand its purpose and benefits, which increases their willingness to seek the service even in the absence of a direct medical request [6].

Another significant associated factor was mothers who had a previous delivery with a congenital malformation were nearly three times more likely to have an antenatal ultrasound examination (AOR = 2.906; 95% CI: 1.222–6.91). This may be due to increased concern about fetal health, heightened risk perception in subsequent pregnancies, and a stronger desire for early detection of possible abnormalities. Prior adverse pregnancy outcomes may also encourage mothers to be more vigilant, adhere closely to ANC recommendations, and seek reassurance through ultrasound examinations, which is supported by evidence in the previous study [30].

### 4.1. Limitation of Study

This study was limited to public health institutions and did not include mothers who delivered in private health facilities, appreciating the discrepancies between private and public sectors. In addition, variables like partner involvement were not considered in this study.

### 4.2. Conclusion and Recommendation

#### 4.2.1. Conclusion

Based on the present study, the proportion of antenatal ultrasound utilization was lower than FIGO and WHO recommendations, which suggest that all pregnancies should undergo a minimum of two scans throughout pregnancy. Significant factors of antenatal ultrasound utilization were residence, women′s knowledge of ultrasound, and congenital malformation.

#### 4.2.2. Recommendation

Based on the findings of this study, the following recommendations are proposed:

The Durame Town Health Office is recommended to enhance the availability and accessibility of antenatal ultrasound services, especially in rural and underserved areas. Monitor and evaluate the utilization of antenatal ultrasound to identify gaps and improve service delivery.

Health professionals (doctors, midwives, and nurses) are encouraged to provide routine counseling to pregnant women on the benefits, purpose, and timing of antenatal ultrasound. Pay special attention to mothers with limited knowledge, prior adverse pregnancy outcomes, or congenital malformations. Encourage early and regular antenatal visits to maximize the uptake of recommended scans.

Program designers/community health programs are encouraged to develop and implement community‐based education programs to raise awareness about the importance of antenatal ultrasound. Target interventions for rural communities and mothers with low knowledge levels. It is recommended to higher education institutions to support efforts to increase utilization of antenatal ultrasound through community outreach programs, health education initiatives, and evidence‐based research.

**Nomenclature**
ANC: antenatal care
AOR: adjusted odds ratio
CI: confidence intervals
COR: crude odds ratio
FMOH: Federal Ministry of Health
LMIC: low‐ and middle‐income country
PNC: postnatal care
U/S: ultrasound
USAID: United States Agency for International Development
WHO: World Health Organization

## Author Contributions

Abdulaziz Assefa, Yohannes Fikadu Geda, Mulugeta Animaw, Seid Jemal, Mangistu Abera, and Aynalem Belay conceived the study and undertook the statistical analysis. Abdulaziz Assefa, Fikremariam Endeshaw, Aynalem Belay, Seid Jemal, Mangistu Abera, and Ayele Sahile supervised the study design and statistical analysis. Interpretation of data and drafting of the manuscript were undertaken by Ayele Sahile, Fikremariam Endeshaw, Yohannes Fikadu Geda, and Aberash Beyene Derribow. Abdulaziz Assefa, Yohannes Fikadu Geda, Mulugeta Animaw, Seid Jemal, Mangistu Abera, and Aynalem Belay supervised proposal development, data collection, data analysis, and interpretation. Abdulaziz Assefa, Yohannes Fikadu Geda, Fikremariam Endeshaw, Ayele Sahile, and Aberash Beyene Derribow reviewed the draft manuscript for intellectual content and participated in its revision. All authors made substantial contributions to the work, critically reviewed the manuscript, approved the final version for publication, and agreed to be accountable for all aspects of the work.

## Funding

No funding was received for this manuscript.

## Disclosure

All authors read and approved the submitted version of the manuscript.

## Ethics Statement

Ethical clearance was obtained from Wolkite University, College of Medicine and Health Sciences, Ethical Review Committee (ERC). A formal letter for permission and support was written to the zonal health department of Durame from Wolkite University, official permission to undertake the study was obtained, and permission to conduct the study was asked. The respondents were informed about the objective, purpose, risks, and benefits of the study and the right to refuse to participate, and then informed written and signed consent was taken. Moreover, the confidentiality of information was guaranteed by using code numbers rather than personal identifiers and by keeping the data locked.

## Consent

The authors have nothing to report.

## Conflicts of Interest

The authors declare no conflicts of interest.

## Data Availability

The data that support the findings of this study are available on request from the corresponding author. The data are not publicly available due to privacy or ethical restrictions.
